# A Systematic Comparison of Perceptual Performance in Softness Discrimination with Different Fingers

**DOI:** 10.3758/s13414-020-02100-4

**Published:** 2020-07-19

**Authors:** Aaron C. Zoeller, Knut Drewing

**Affiliations:** Department of General Psychology, Justus-Liebig University Giessen, Building F1, Room 307, Otto-Behaghel-Str. 10F, D-35394 Giessen, Germany

**Keywords:** Psychophysics, Perception, Softness discrimination, Active exploration

## Abstract

In studies investigating haptic softness perception, participants are typically instructed to explore soft objects by indenting them with their index finger. In contrast, performance with other fingers has rarely been investigated. We wondered which fingers are used in spontaneous exploration and if performance differences between fingers can explain spontaneous usage. In Experiment 1 participants discriminated the softness of two rubber stimuli with hardly any constraints on finger movements. Results indicate that humans use successive phases of different fingers and finger combinations during an exploration, preferring index, middle, and (to a lesser extent) ring finger. In Experiment 2 we compared discrimination thresholds between conditions, with participants using one of the four fingers of the dominant hand. Participants compared the softness of rubber stimuli in a two-interval forced choice discrimination task. Performance with index and middle finger was better as compared to ring and little finger, the little finger was the worst. In Experiment 3 we again compared discrimination thresholds, but participants were told to use constant peak force. Performance with the little finger was worst, whereas performance for the other fingers did not differ. We conclude that in spontaneous exploration the preference of combinations of index, middle, and partly ring finger seems to be well chosen, as indicated by improved performance with the spontaneously used fingers. Better performance seems to be based on both different motor abilities to produce force, mainly linked to using index and middle finger, and different sensory sensitivities, mainly linked to avoiding the little finger.

## Introduction

In everyday life, haptic perception is essential for interacting with our surroundings, because it allows us to identify and discriminate relevant properties of objects that cannot really be distinguished by other senses. The compliance of an object is one important property that can be determined most exactly through haptic exploration (Lederman & Klatzky, 1987). Its perceptual correlate is softness. To assess softness, we use specifically optimized exploratory movements. For example, when we judge the ripeness of an avocado in the supermarket, or how comfortable a cushion is, we indent the surface of that object to gather softness information (Lederman & Klatzky, 1987). In typical studies on softness perception, participants are constrained to explore objects using only the index or only the middle finger (e.g., Friedman et al., 2008; Fujita & Ohmori, 2001; Kaim & Drewing, 2011; Srinivasan & LaMotte, 1995). However, it seems that in less constrained situations humans prefer to use several fingers to perceive softness (Katz, 1925; Pense-Lheritier et al., 2006). Here, we investigate in three experiments if there is a systematic selection of spontaneously used fingers and on which factors the selection might be based. We know that fingers differ in the ability to produce force and the size of the finger tip, which both potentially influence the performance in softness perception (Kaim & Drewing, 2011; Nicholson, Maher, & Adams, 1998; Srinivasan & LaMotte, 1995). We wondered if performance differences between fingers can explain patterns of spontaneous usage.

We investigate softness in objects varying in compliance, which is defined as the extent of object deformation under a given force (mm/N). Humans typically explore the softness of a compliant object manually by repeatedly indenting the object’s surface over time while it is in a fixed position (Lederman & Klatzky, 1987). For this purpose, different types of behavior like pressing into the surface (Kaim & Drewing, 2011), squeezing an object (Tan Durlach, Beaurgard, & Srinivasan, 1995) or tapping on the object (Friedman et al., 2008; Higashi, Okamoto, Yamada, Nagano, & Konyo, 2018) are performed. Recent studies examining softness perception investigated exploration behavior in different settings: participants used tools (LaMotte, 2000), had a minimum of constraints on their finger force and velocity (Kaim & Drewing, 2011), were free to choose the number of indentations and changes between stimuli in discrimination (Lezkan, Metzger, & Drewing, 2018) or were presented with prior knowledge about the stimuli (Lezkan, Metzger, & Drewing, 2018; Zoeller et al., 2019). However, an important feature of natural active exploration was broadly neglected. When people spontaneously explore the softness of an object, they tend to use several different and sometimes multiple fingers (Katz, 1925; Pense-Lheritier et al., 2006). However, in studies to date, participants have typically been instructed to only use their index finger in single-finger indentation (e.g., Higashi et al., 2018; Fujita & Ohmori, 2001; Kaim & Drewing, 2011; Lezkan, Metzger, & Drewing, 2018; Srinivasan & LaMotte, 1995; Saig et al., 2012; Zoeller et al., 2019).

An exception to this neglect of the study of natural exploration can be seen in studies investigating the specific exploratory behavior of squeezing, in that object softness is typically explored with a pinch grip using thumb and index finger (Di Luca, 2011; Di Luca & Ernst, 2014; Freyberger & Färber, 2006; Tan et al., 1995). The role of the fingers used in a pinch grip is not only to gather sensory information, but also to stabilize the explored object (Di Luca & Ernst, 2014). Results indicate that information from both thumb and index finger is used with unequal weights (Di Luca, 2011). However, no statement on a potential role for fingers other than thumb and index finger in natural softness perception can be made from these studies, and the pinch grip is a special case in that it is only applicable in situations in which the explored object is small enough to be grasped (Lederman & Klatzky, 1987). Katz (1925) studied natural exploration behavior with all fingers in situations that did not include the motor requirements of a pinch grip. He reported that all participants spontaneously used at least index and middle fingers − together or in alternation − to examine a given stimulus. These early results suggest that humans systematically use several fingers in haptic exploration, which might be a more efficient strategy as compared to only using one finger. Drewing (2014) showed that for objects with high compliance, softness discrimination is better when participants used index, middle, and ring fingers as compared to the index finger only, suggesting that the use of different fingers can improve perceptual performance as indicated by the spontaneous usage found in Katz (1925). Still, from these studies it is not clear how performance in softness perception differs between the fingers and why certain fingers may be preferred.

If humans explore optimally in natural situations, we would expect a preference for using fingers that show better perceptual performance. We would also expect the use of multiple fingers, because this should enhance performance as compared to a single finger by the simultaneous or sequential integration of softness information from different fingers (Di Luca, 2011; Drewing, 2014). Integration processes can lead to a more precise representation due to fusion of sensory data from different information sources, combined with a higher weighting of more precise sources (Ernst & Banks, 2002; Drewing & Ernst, 2006; Lezkan, Metzger, & Drewing, 2018).

But why should fingers differ in their perceptual precision? It is known that fingers differ in characteristics that likely influence the gathering of sensory information in active softness perception. Fingers have been demonstrated to differ in their motor ability in experiments that explored grip strength as well as when measuring produced forces for each finger individually: middle and index fingers were stronger than ring and little fingers, and the little finger was the weakest overall (Li, Latash, & Zatsiorsky, 1998; MacDermid et al., 2004; Talsania & Kozin, 1998; Quaine, Vigouroux, & Martin, 2003). Force production likely affects a finger’s ability to effectively generate object deformations that serve as cues to object softness.

It has also been suggested that fingers differ in the sensory ability to perceive pressure, spatial acuity, and in their two-point discrimination threshold (Dellon, Mourey, & Dellon, 1992; Louis et al., 1984; Manser-Smith et al., 2018; Sathian & Zangaladze, 1996; Schweizer et al., 2000; Vega-Bermudez & Johnson, 2001; Weinstein, 1968). However, previous results are somewhat heterogeneous. In some studies investigating point localization and two-point discrimination, a better performance for thumb, index, and middle finger as compared to ring and small finger in the glabrous skin was found (Manser-Smith et al., 2018). The little finger seems to have the worst localization threshold (Louis et al., 1984; Schweizer et al., 2000). Similar patterns have also been found in active and passive volume perception (Zhang et al., 2018; Zhang et al., 2019). In contrast, Dellon, Mourey, and Dellon (1992) found better performance of the little finger in two-point discrimination as compared to the index finger.

In two studies investigating spatial acuity, no difference in spatial acuity between index, middle, and ring fingers but a lower spatial acuity for the little finger was found (Ducan & Boynton, 2007; Sathian & Zangaladze, 1996). On the contrary, Vega-Bermudez and Johnson (2001) found declining spatial acuity from the index to the middle to the ring finger, not investigating the thumb or little finger.

Previous results mostly indicate a declining sensory sensitivity from the index to the little finger, with some exceptions. Differences in the sensitivity of different fingers might be partly related to differences in the spatial density of mechanoreceptors (Dillon, Haynes, & Henneberg, 2001; Gellis & Pool, 1977; Peters, Hackeman, & Goldreich, 2009). Further, sensory sensitivity of the different finger tips correlates with the cortical representation of fingers in S1 and Brodmann 3b and 1, indicating the neuronal basis for systematic differences between fingers (Ducan & Boynton, 2007).

Differences between fingers in one of the factors – the ability to produce force and sensory ability – could lead to performance differences in softness exploration and thus explain differences in usage frequency (Katz, 1925). In this study comprised of three experiments, we investigate which fingers are used in spontaneous softness discrimination, and if patterns of usage can be explained by performance differences resulting from differences in sensory sensitivity or the ability to produce force. In Experiment 1 we investigated spontaneous behavior. Participants discriminated two rubber-disc stimuli while exploration movements were filmed. We divided each exploration into sequences of events (“exploration phases”) and analyzed the type of movement and the fingers used. In Experiment 2 we investigated if spontaneous finger usage relates to performance differences. We used a two-interval forced choice (2IFC) discrimination task and the method of constant stimuli to compare softness discrimination performance of index, middle, ring, and little fingers in single-finger exploration. Participants explored two rubber stimuli with unconstrained force and had to judge which one was softer. We measured just noticeable differences (JNDs) as 84%-discrimination thresholds, peak indentation forces, and finger width. In Experiment 3 we examined performance in softness discrimination for the four fingers with constant forces to dissociate the influences of finger strength and sensory sensitivity on finger performance. Therefore, we prescribed constant forces (10.0–15.0 N) and again measured JNDs, peak indentation forces, and finger width.

## Experiment 1

In the first experiment we investigated spontaneous exploration behavior to examine how well the method of single-finger indentation with the index finger that is broadly instructed in scientific studies can represent a natural exploration process. Motivated by the observations of Katz (1925), we hypothesized that to explore the softness of objects people spontaneously use several different fingers both sequentially and simultaneously, and that they prefer the index and the middle finger.

Eighty-three participants compared the softness of two silicon rubber stimuli using the fingers of their dominant hand. Each participant performed only one trial. During the exploration we filmed the fingers of the participants. We analyzed the number of the fingers used, which fingers were used sequentially and simultaneously, the number of exploration phases (a new exploration phase starts with a change in the fingers used or in the exploration behavior), and the types of exploration behavior (like indenting, lateral motion, and tapping).

### Method

#### Participants

Eighty-three healthy students from the Justus-Liebig-University in Giessen participated in this study (27 males). The data collection was done in the cafeteria of the university. People who reported impairments in motor control, or in sensory functions of their hand, and people who had more or fewer than five fingers were excluded from the study. Participants were naïve to the purpose of the study, and they gave written informed consent. Methods and procedures of all three experiments of the study were approved by the local ethics committee LEK FB06 at Giessen University, and in accordance with the ethical standards laid down in the 2013 Declaration of Helsinki.

#### Stimuli and setup

**Fig. 1:**
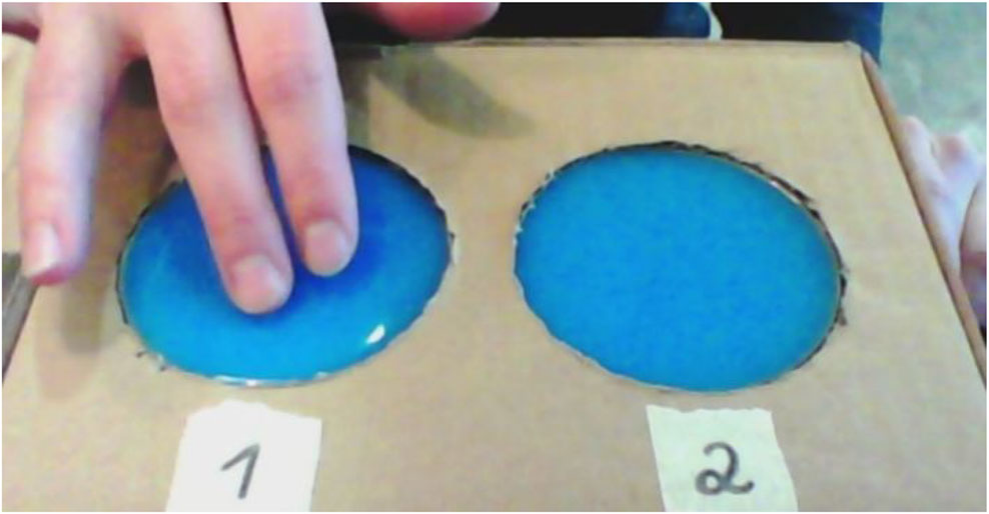
Screenshot of the camera filming a participant exploring the stimuli of Experiment 1

We used two silicone rubber disc stimuli of similar appearance with a height of 38 mm and a diameter of 75 mm. The compliances of the two stimuli were 0.44 mm/N and 0.48 mm/N. We measured the compliance of the stimuli by repeatedly indenting them with a flat-ended cylindrical probe (1 cm^2^ area) and fitting regression lines of indentation depth on force data; the slope of the regression line then estimates compliance (see Kaim & Drewing, 2011, for details of the production and measurement process). The difference between the two present stimuli (0.04 mm/N) is equivalent to half a Weber fraction in softness discrimination (Kaim & Drewing, 2011). This difference was chosen to be very small (hardly distinguishable) to ensure an extensive exploration process. Because we were interested in exploration behaviors not including the pinch grip, both stimuli were embedded in a box, to exclude grasping and squeezing behavior. Stimuli were marked with the numbers one (0.44 mm/N) and two (0.48 mm/N) to be easily identifiable in later analyses (Fig. 1). No chinrest was used, so the viewing distance varied across participants. In Experiment 1, no blindfolds were given during the experiment, allowing participants to receive and use visual feedback to guide their exploratory behavior, as is the case in most natural situations. As shown in Cellini et al. (2013), this might have an impact on the perceptual performance. However, in Experiment 1 we did not investigate perceptual performance, but natural exploratory behavior, which would be potentially strongly perturbed by blindfolding our unpracticed participants.

We recorded the exploration process with an HD 720p camera (CREATIVE Live! Cam Sync HD, spatial resolution 1,280 x 720 pixels, temporal resolution 30 Hz). The scene was filmed in an angle of approximately 60° and the distance between camera and stimuli was approximately 30 cm.

#### Procedure and design

Participants performed only a single trial to ensure exploration behavior was spontaneous and natural. They were instructed to discriminate the compliance of the two given stimuli and point to the softer one. Participants were also instructed to only touch one stimulus at a time and to only use their dominant hand. No further constraints or instructions on exploratory behavior were given. Participants could start with either stimulus, change between stimuli, and touch the surfaces as often as desired. After the camera was turned on, participants were told to start the exploration. Participants were asked to point to the stimulus that was perceived as softer.

#### Data analysis

Exploration behavior of each participant was divided in sequential phases. A new phase was defined by the start of the exploration; when exploration behavior changed (e.g., tapping, indenting, lateral motion); or the finger used was changed (e.g., changing from the middle to the ring finger, or adding or removing a finger while exploring). We used the Exploratory Procedures (EPs) defined by Lederman and Klatzky (1987) supplemented with the considerations of Friedman et al. (2008) to categorize different types of behavior. We discriminated between two movement schemes falling under the EP *Pressure*, namely tapping (very short contact between stimulus and finger, fast movement) and indenting (longer duration, slower movement), as they are suggested to be two distinct types of exploration behavior used to explore softness (Friedman et al., 2008; Higashi et al., 2018). Behavioral data was coded by two raters to test inter-rater reliability, who used a small number of examples to decide how to categorize unclear behavior. We analyzed the number of phases during the process and the performed exploration behavior for every participant. Normalized by the number of phases for each participant, we calculated the percentages of exploration phases that involved each specific finger, and the percentages of exploration phases that involved finger combinations.

To further compare the differences in usage of fingers, we calculated the individual arcsine transformed percentages of phases involving each single finger and the percentages of phases involving different numbers of fingers based on the averaged values of the coding of rater 1 and rater 2 for each participant. We then performed a repeated-measures ANOVA with the within-participant variable NumberOfFingers (One, Two, Three, Four, Five) and a repeated-measures ANOVA with the within-participant variable Finger (five levels: Thumb, Index, Middle, Ring, Small) followed by Holm-Bonferroni-corrected paired *t*-tests for each level.

### Results

On average, participants responded with 54% accuracy, which is not significantly different from the 50% chance level in a two-choice task (binomial test: *p* = .260, one-sided). The result shows that the chosen stimuli were indistinguishable during spontaneous exploration, which ensured a careful exploration during the task. The inter-rater reliabilities for behavior categorization and for finger usage were both “almost perfect,” *κ* = .812, and *κ* = .863, respectively (Landis & Koch, 1977). The correlation between both raters regarding the number of executed phases was *r* = .621, *p* < .001. Results are presented as the average values between both raters. On average participants performed 3.4 different sequential phases during the exploration process of both stimuli. Individual data ranged from one to ten phases.

Participants predominantly indented the stimuli during exploration. Ninety-five percent of all participants used this behavior, 70% of all participants even exclusively indented the stimuli. Lateral motion was performed by 26% of all participants, but only 5% exclusively performed lateral motion. Only 6% of all participants showed tapping behavior and no participant exclusively showed tapping behavior. Twenty-five percent of all participants showed more than one behavior, always including indentation.

**Fig. 2:**
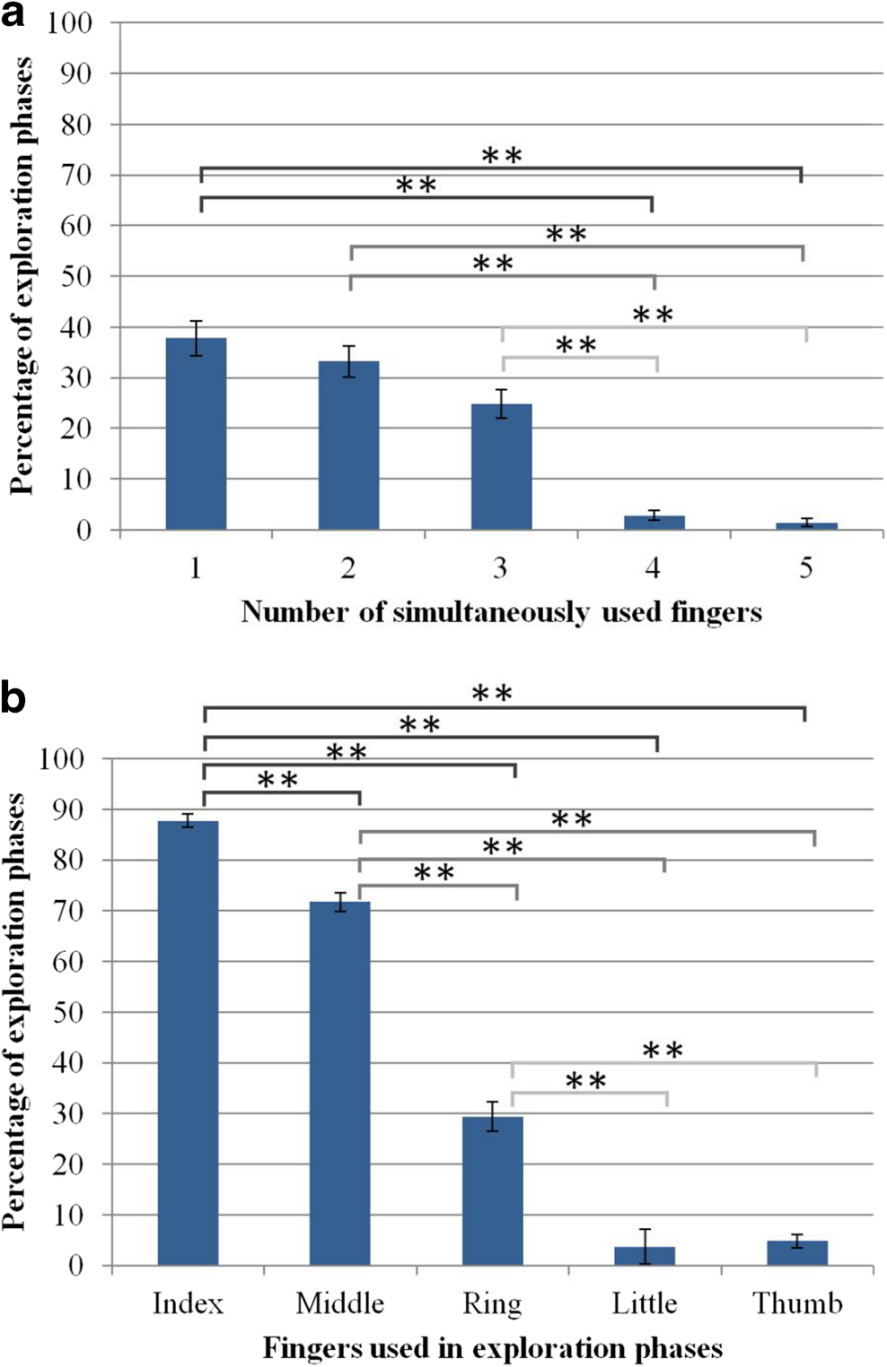
(**A**) Percentage of exploration phases including different numbers of simultaneously used fingers. (**B**) Percentages of exploration phases including different fingers (sum > 100%). Significant effects are marked by asterisks (* = *p* < .05; ** = *p* < .01)

In 38% of the exploration phases only a single finger was used to explore the objects. In 33% of the phases, participants explored with two fingers simultaneously, and in 25% three fingers were used simultaneously (Fig. 2 A). Thus, all three behaviors seem to be common, whereas only very rarely were four or five fingers used simultaneously (3% and 1%). We found a significant effect in the ANOVA comparing the number of fingers used, *F*(4, 328)= 41.91, *p* < .001, *η*_*p*_^*2*^ = 0.338. In the Holm-Bonferroni-corrected *t*-tests we found no significant difference between the percentages of usage of one, two, or three fingers simultaneously, and no significant differences between the percentage of usage of four or five fingers simultaneously (*p* > .05, *d* < 0.232). However, we found significant differences between the percentages of using one, two, and three fingers simultaneously as compared to using four or five fingers (*p* < .001, *d* > 0.908). Results indicate that participants used one, two, and three fingers more frequently at the same time as compared to four and five fingers at the same time.

Despite the frequent appearance of single-finger indentation, only 7% of the participants used just one finger during the whole exploration process. All other participants (93%) alternated between multiple fingers or used them simultaneously, indicating a natural use of multiple fingers during softness exploration. Twenty-four percent of participants used a single finger, two fingers, and three fingers simultaneously in different phases. Twenty-one percent of the participants used a single and two fingers simultaneously, 13% used two and three fingers simultaneously, and 11% used only a single finger (but with varying fingers in different phases). All other combinations were used by less than 10% of the participants.

There were also preferences for specific fingers. The index finger was used in 88% and the middle finger in 72% of all exploration phases. The ring finger was used in 29%, and little finger and thumb in 4% and 5% of all phases (Fig. 2 B). We found a significant effect in the ANOVA comparing the usage of each finger *F*(4, 328) = 296.40, *p*< .001, *η*_*p*_^*2*^= 0.783. In the Holm-Bonferroni-corrected t-tests we found significant effects for all comparisons (*p*_*holm*_< .001, *d*> 520), except when comparing the little finger to the thumb, *t*(82) = 1.14, *p*_*holm*_ = 1.000, *d* = 0.13. Results indicate a significantly descending percentage of usage from index finger to little finger. The usage of small finger and thumb did not differ.

Over the whole experiment, 100% of the participants used the index finger in any exploration phase, 93% of the participants used the middle finger, 59% the ring finger, 16% the thumb, and 9% the little finger in any exploration phase.

When single-finger exploration was performed, participants used mainly the index finger (71% of the phases), but also the middle finger (23%; other fingers < 6%). In two-finger exploration they used a combination of index and middle finger in 96% of the phases and a combination of middle and ring finger in 4% of all phases. In a single phase a combination of index finger and thumb was used. When participants explored stimuli with three fingers, a combination of index, middle, and ring finger was used in 94% of the phases and a combination of index finger, middle finger, and thumb in 6% of all phases.

### Discussion

We investigated natural exploration behavior in softness discrimination tasks to examine which fingers and combinations are spontaneously used. We observed that participants explore in different phases that include varying numbers and combinations of fingers. They preferred to use one, two, or three fingers simultaneously as compared with four or five fingers. In line with Katz (1925), index and middle fingers were used most frequently, followed by the ring finger. Other fingers were mostly avoided.

The results further show that indenting the stimulus is the predominant exploration type for exploring softness, which is in line with Lederman and Klatzky (1987). However, lateral motions across the surface of the stimuli were also performed, which Lederman and Klatzky (1987) had observed to be the exploratory procedure to investigate the texture of an object’s surface. Here, this behavior might be related to the exploration of surface softness, as it is performed when judging the softness of cloth (Pense-Lheritier et al., 2006). The rare appearance of tapping behavior can be explained by the properties of our stimuli. Tapping is mostly performed when the compliance of the explored object is very low, such that indentation does not seem possible (Higashi et al., 2018). However, the stimuli we used were fairly compliant, and hence participants might have indented the stimuli rather than tapping them. In Experiments 2 and 3 we focus on indentation behavior because it is the behavior predominantly used to explore the compliance of objects.

The results also show that the index finger is used most frequently for exploration and that single-finger exploration with the index finger is a substantial part of the exploration behavior (27%), but insufficient to represent the complete exploration process (as implicitly concluded in previous studies). Ninety-three percent of our participants used multiple fingers sequentially and simultaneously during the exploration, most often the index, middle, and ring fingers. We hence speculate that the use of different fingers might be superior to exploration with only one finger. The integration of information from different fingers, both simultaneous and sequential, might reduce noise and enhance performance (Ernst & Banks, 2002; Drewing & Ernst, 2006; Lezkan, Metzger, & Drewing 2018). It appears feasible that participants predominantly use fingers that deliver the most precise information, suggesting that the high usage frequency of index and middle finger (and ring finger) is because of better perceptual performance of these fingers.

These suggestions are also paralleled by differences in the factors of strength and sensory sensitivity. Differences in grip and press strength show that the middle and index fingers are stronger than the ring and little fingers, whereas the little finger is the worst (Li, Latash, & Zatsiorsky, 1998; MacDermid et al., 2004; Talsania & Kozin, 1998; Quaine, Vigouroux, & Martin, 2003). Additionally, results from Louis et al. (1984), Manser-Smith et al. (2018), and Schweizer et al. (2000) indicate a declining spatial acuity from index and middle fingers to the ring finger and to the little finger. The results are also in line with Zhang et al. (2018, 2019), who found a better performance for index and middle fingers as compared to the ring and small fingers in volume perception. Both factors – the ability to produce force and sensory ability – potentially influence the performance in softness perception and might thus explain the higher frequency of usage.

## Experiment 2

In the second experiment we systematically compared performance in softness discrimination of different single fingers (index, middle, ring, or little finger), using a 2IFC discrimination task and the method of constant stimuli. The thumb was not included. When indenting the surface of the stimuli with index, middle, ring, or small finger the participant can remain in the same hand/arm position. When indenting the stimulus with the thumb, this position needs to change completely, which potentially influences perception. Additionally, in the experimental setup, a very unnatural hand position would be necessary to prevent the hand from interfering with the force-feedback device’s arm. This would also influence the collected data leading to a dataset hardly comparable with the other four fingers. We hypothesized that participants were able to discriminate softness better with the most frequently spontaneously used index and middle fingers as compared to the ring and little fingers. We also expected a higher performance for the ring finger as compared to the little finger, following the results of Experiment 1. In every trial participants used one finger to judge which of two stimuli was softer; they were allowed to indent the surface of each stimulus only once with unconstrained force. In this case both differences in sensory sensitivity and finger strength influence performance. We measured just noticeable differences (JNDs) as 84%-discrimination thresholds for each finger. We additionally measured peak indentation forces and finger width to estimate differences in produced forces and in contact area.

### Methods

#### Participants

To estimate the number of participants needed in Experiments 2 and 3, we performed a power analysis using G*Power 3.1 (Faul et al., 2007). We calculated the expected effect size (f = 0.40) from Drewing (2014), which compared one- versus three-finger indentations, but is to our knowledge the only study comparing performance using the index, middle, and ring finger within one sample. We set the probability of an α error = .05 and the Power (1- probability of a β error) = .95, resulting in a sample size of 15. Due to the design of the experiments (we used 4 × 4 Latin squares, to account for repetition effects), we used a sample size of 16 participants in both Experiment 2 and Experiment 3.

Sixteen healthy students from the Justus-Liebig-University in Giessen participated in Experiment 2 (14 females, two males, average age 25 years, range: 20–34). All participants reported being right-handed. The first language of all participants in Experiment 2 and 3 was German and both experiments were conducted completely in German. Participants reported no tenosynovitis and motor impairments in the past and were tested for sensory impairments using a 2-Point discriminator (by Exacta). All participants had a discrimination threshold of 4 mm or better on any finger. All participants had normal or corrected-to normal vision. Participants received 8€ per hour for participating and were naïve to the purpose of the study. All participants gave informed written consent.

#### Stimuli and setup

**Fig. 3:**
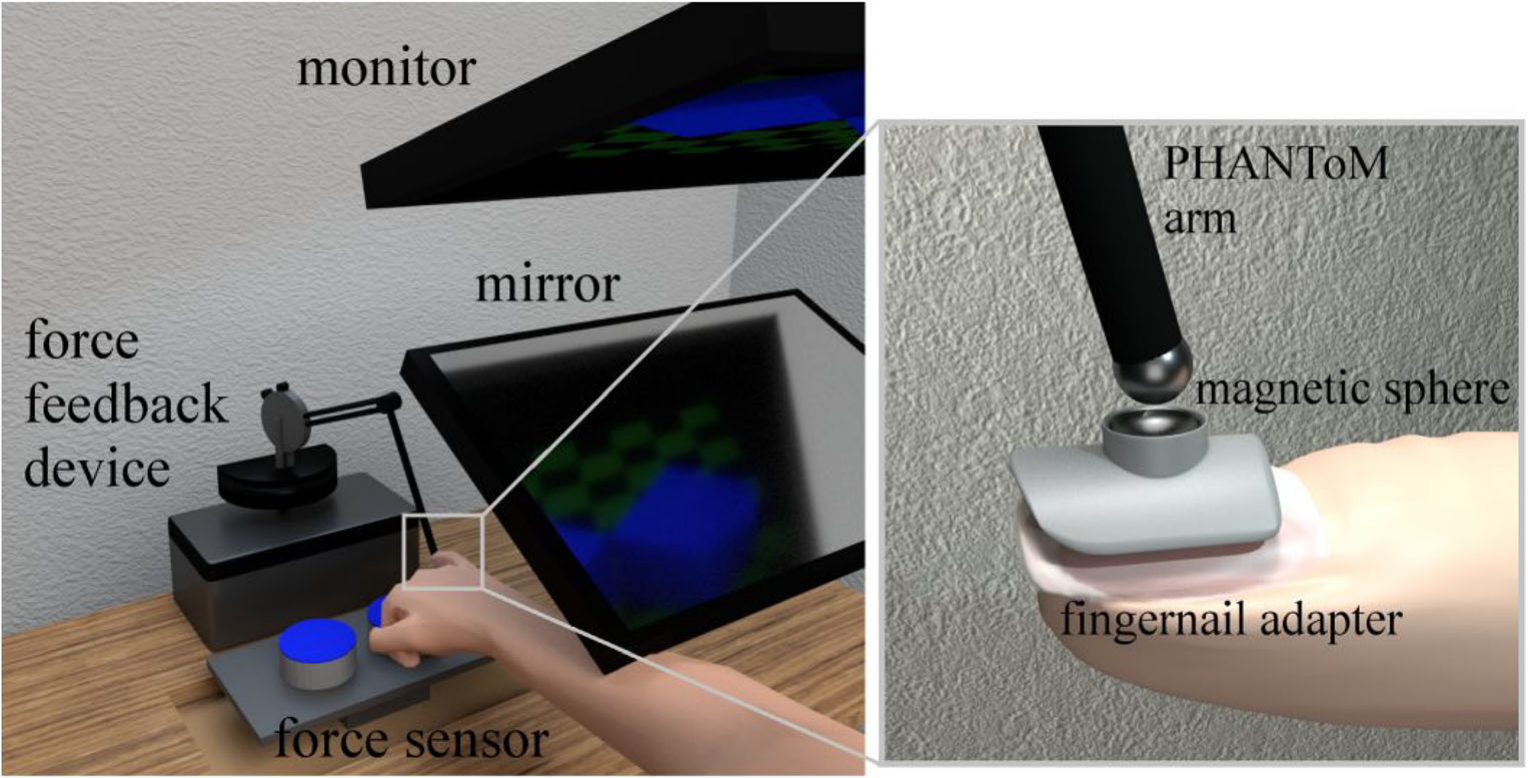
Scheme of the visuo-haptic workbench and magnetic finger nail adapter

Participants performed the task on a custom-made visuo-haptic workbench containing a 24-in. 3D screen (120 Hz, 1,600 × 900 pixels), a force sensor (resolution 0.05 N, temporal resolution 682 Hz) to collect data of executed finger force, and a PHANToM 1.5A haptic force feedback device (spatial resolution: 0.03 mm, temporal resolution: 1,000 Hz) to collect finger position data (Fig. 3). To allow a maximum amount of freedom in finger movements for all axes in the PHANToM’s 38 × 27 × 20 cm^3^ workspace, the right index finger of participants was connected to the device via a spherical magnetic adapter. The adapter was fixed to the fingernail and left the finger tip free, to allow for bare-finger exploration. To present a 3D scene we used stereo glasses (Nvidia 3D Vision 2). Participants looked at the screen through a front surface mirror (viewing distance 40 cm) aligned with the haptic scene to ensure a natural connection between haptic action and visual feedback and to eliminate real visual feedback. The finger position was displayed via a green sphere (3 mm) in the virtual scene. During contact with the stimuli the sphere disappeared to give no visual feedback about the stimulus deformation in order to prevent any potential influence of visual information on the haptic perception thresholds (Cellini et al., 2013). A chinrest was used to stabilize the heads of participants. All devices were connected to a PC where custom-made software controlled the experiment and data collection. Audio signals were given via stereo headphones (Sennheiser HD 280 Pro).

We used 12 cylindrical shaped silicone rubber stimuli. Two equally compliant stimuli^1^ (compliance: 0.39 mm/N) were used as standards. The remaining ten stimuli (five softer than the standards, and five harder) were used as comparison stimuli to test discrimination behavior. Both standards were also compared to each other. Compliance of the stimuli ranged from 0.25 mm/N to 0.55 mm/N and differed between two consecutive softness levels by approximately 0.03 mm/N, so that compliance levels span approximately 2.5 Weber-fractions around the standard stimuli. The value for the Weber fraction in softness perception of 15% is taken from Kaim and Drewing (2011).

#### Procedure and design

The experiment included four different conditions. In each condition only one of the four fingers (index, middle, ring, and little) of the dominant hand was used to explore the given objects. In each trial one standard and one comparison stimulus were placed side by side and participants had to judge which one was softer. We used a 2IFC discrimination task combined with the method of constant stimuli to assess JNDs by 84%-discrimination thresholds. Each of the pairs of standard stimulus and comparison was presented 10 times (20 times for both versions of the standard). However, due to a technical mistake, the number of comparisons between the two standard stimuli was unintentionally doubled (40 times), so that participants performed overall 240 trials per condition.

In the beginning of each trial one of the two stimuli was presented in the visual scene to define the starting position for the exploration. Starting positions were presented in a randomized order, with left or right occurring equally often. Afterwards an auditory beep signaled the start of the exploration phase and participants indented the surface of the starting stimulus. Participants were instructed to explore the objects by pressing into the surface and to indent each of the two stimuli only once. When touching the starting stimulus, a visual representation of the second stimulus appeared. After both stimuli had been explored, the word “softer” appeared above each stimulus and participants had to indicate by a virtual button press which of the stimuli they had perceived to be softer. After each trial, participants moved away from the stimuli. The experimenter changed stimuli and started the next trial manually. No feedback on performance was given.

The order of the four conditions was balanced across participants by using a 4 × 4 Latin square. The experiment was divided into two sessions, which contained two of the four conditions each. Every condition contained 240 trials, so that every participant performed 960 trials in total. Each condition was subdivided in five blocks (each 48 trials). Between every block a break of 1 min was implemented to counteract fatigue. Before starting with the experimental trials, participants performed eight test trials to familiarize with the task. In the beginning of the experiment, the finger widths of each participant were measured at the midpoint of the maximal difference between the two curves described by lunula and eponychium of each finger. On average, the experiment took 6 h in total.

#### Data analysis

We used force and position data to assess the number of indentations and the indentation forces per stimulus and trial. Force data was smoothed by a moving-averaging window with a kernel of 45 ms. To find force maxima, we identified turning points in which the derivative of force over time changed from positive to negative. A force maximum was considered to represent a separate indentation movement, when the temporal distance to the next force maximum was at least 180 ms. By this criterion we avoided misinterpretation of small local force variation, for example from the little finger shaking during release. Indentations were attributed to one of the two stimuli based on the finger position data. Trials with more than one indentation per stimulus were defined as invalid trials and were excluded from later analysis. We then determined individual psychometric functions for each of the four conditions as the percentages of trials in which the comparison stimulus was perceived to be softer than the two standard stimuli. When comparing the two standard stimuli, each stimulus was assigned to be the comparison stimulus in half of the trials, as defined by the experimental script. We fitted cumulative Gaussian functions as a function of stimulus compliance using Bayesian methods in psignifit4 toolbox (Schütt et al., 2016), where μ assessed the point of subjective equality (PSE) and the parameter σ of the cumulative Gaussian function was taken to assess the just noticeable difference (JND) as the 84%-discrimination threshold (cf. Helbig & Ernst, 2007; Zhang et al., 2018). Laps rate and guessing rate were set to zero. Individual JNDs were entered in a repeated-measures ANOVA with Finger (Index, Middle, Ring, Little) as within-participant variable. Holm-Bonferroni-corrected paired *t*-tests were used to compare the JNDs in the different conditions. We further compared PSEs and the amount of valid trials between all four fingers in two repeated-measures ANOVAs with Finger (Index, Middle, Ring, Little) as the within-participant variable, and individually averaged peak forces between fingers using another ANOVA. Finally, as an indicator for differences in the contact area during the exploration, we analyzed the individual widths of each finger with an ANOVA.

### Results

On average, 97.3% of the trials were valid and could be used in the analysis (Index Finger: 98.4%; Middle Finger: 98.3%; Ring Finger: 97.1%; Little Finger: 95.5%). The amount of valid trials did not significantly differ between fingers, *F*(3,45) = 1.76, *p* = .168, *η*_*p*_^*2*^ = 0.105. In the ANOVA comparing PSEs for all four fingers, no significant main effect was found, as expected (*F*(3,45) = 0.54, *p* = .657, *η*_*p*_^*2*^ = 0.035, overall average; 0.402 mm/N).

**Fig. 4:**
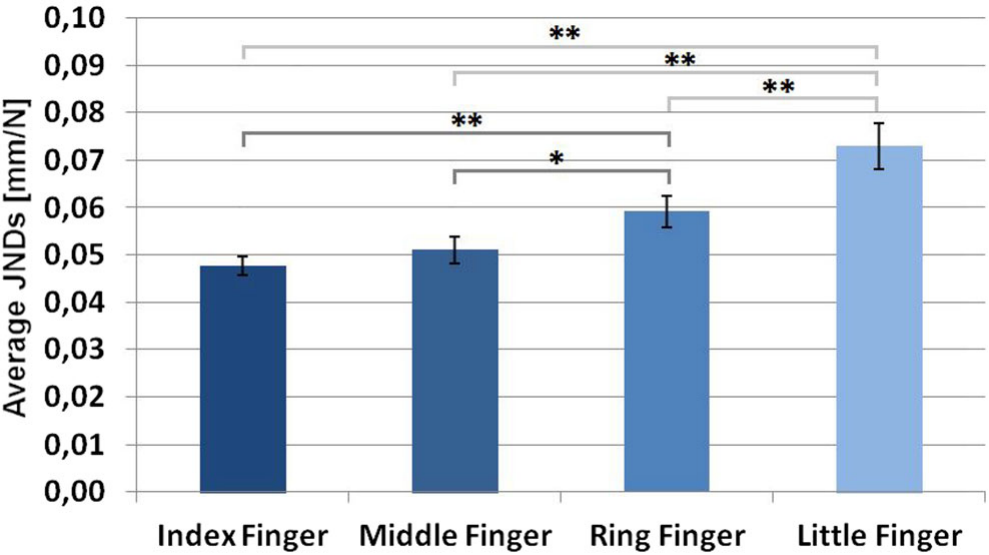
Average just noticeable differences (JNDs; mm/N)] and *SEM* for each finger. Significant effects are indicated by asterisks (* = *p* < .05; ** = *p* < .01)

Comparing JND differences between fingers (Fig. 4), we found a significant main effect, *F*(3,45) = 22.90, *p* < .001, *η*_*p*_^*2*^ = 0.604. We compared different fingers pair-wise by calculating 6 *t*-tests. Although these tests were planned, we controlled for multiple testing by using Holm-Bonferroni-correction. In line with our hypothesis, we found a significantly better performance (lower JND) for the index finger as compared to ring and little fingers, *t*(15) = 3.99, *p*_*holm*_ = .005, *d* = 1.00, and *t*(15) = 5.65, *p*_*holm*_ < .001, *d* = 1.41, respectively. Also in line with our hypothesis, we found increased performance with the middle finger as compared with the ring and little fingers, *t*(15) = 3.29, *p*_*holm*_ = .010, *d* = 0.82, and *t*(15) = 6.66, *p*_*holm*_ < .001, *d* = 1.67, respectively. No significant difference between index and middle fingers was found, *t*(15) = 1.18, *p*_*holm*_ = .258, *d* = 0.29. We found a better performance (lower JND) when using the ring finger as compared to the little finger, in further support of our hypothesis (*t*(15) = 3.90, *p*_*holm*_ = .005 , *d* = 0.97).

**Fig. 5:**
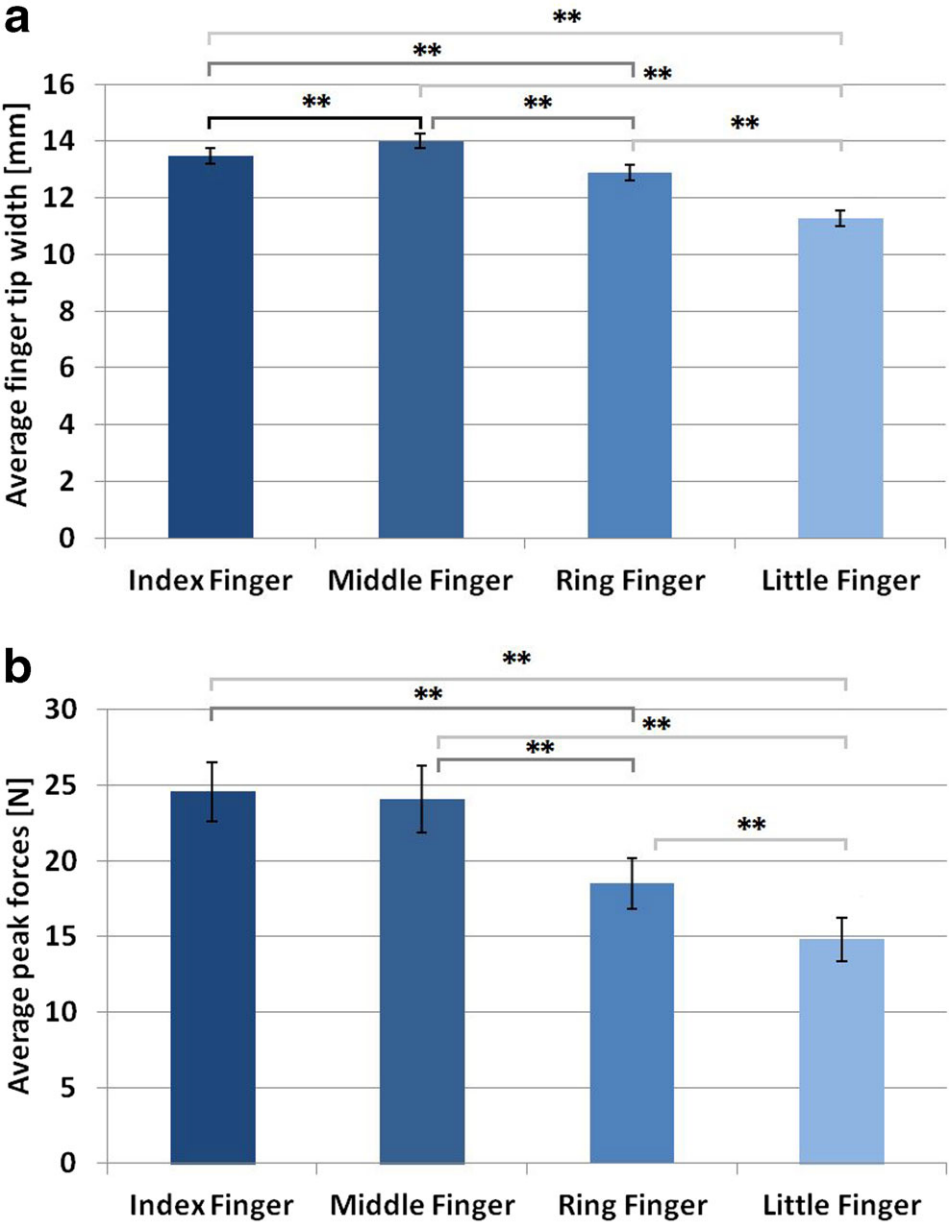
(**A**) Average finger-tip width (mm) and *SEM* for each of the four fingers. (**B**) Average forces (N) and *SEM* for each of the four fingers. Significant effects are indicated by an asterisk (* = *p* < .05; ** = *p* < .01)

We compared the width of the fingers (Fig. 5 A) and peak forces used (Fig. 5 B). Fingers differed significantly in their width, *F*(3,45) = 125.80, *p* < .001, *η*_*p*_^*2*^ = 0.893. Holm-Bonferroni-corrected pair-wise *t*-tests showed that the middle finger was on average wider as compared with index, ring, and little fingers, *t*(15) = 3.81, *p*_*holm*_ = .003, *d* = 0.95, *t*(15) = 9.21, *p*_*holm*_ < .001, *d* = 2.31, and *t*(15) = 20.03, *p*_*holm*_ < .001, *d* = 5.00, respectively. The index finger was on average wider as compared with ring and little finger, *t*(15) = 3.66, *p*_*holm*_ = .003, *d* = 0.92, and *t*(15) = 11.95, and *p*_*holm*_ < .001, *d* = 2.99, respectively. The ring finger was on average significantly wider as compared with the little finger, *t*(15) = 11.38, *p*_*holm*_ < .001, *d* = 2.84.

Peak forces differed significantly between fingers in the ANOVA, *F*(3,45) = 34.21, *p* < .001, *η*_*p*_^*2*^ = 0.695. Paired *t*-tests showed that participants used higher forces with the index finger as compared with ring and little fingers, *t*(15) = 4.66, *p*_*holm*_ < .001, *d* = 1.17, and *t*(15) = 8.16, *p*_*holm*_ < .001, *d* = 2.04, respectively. They also used higher forces with the middle finger as compared with ring and little fingers, *t*(15) = 5.38, *p*_*holm*_ < .001, *d* = 1.35, and *t*(15) = 7.15, *p*_*holm*_ < .001, *d* = 1.79, respectively. We found no difference between index and middle fingers, *t*(15) = 0.45, *p*_*holm*_ = .658, *d* = 0.11, but a difference between ring and little fingers, *t*(15) = 4.94, *p*_*holm*_ < .001, *d* = 1.24.

### Discussion

We systematically compared perceptual performance of the index, middle, ring, and little fingers in an active softness discrimination task. Participants had smaller JNDs when performing the task with index and middle fingers as compared with ring and little fingers. Additionally, JNDs were smaller when using the ring finger as compared with the little finger. The results are in line with our hypothesis: the better performance with index and middle fingers can well explain why in spontaneous exploration index and middle fingers are used most frequently, as found in Experiment 1 and by Katz (1925), if assuming that humans tend to optimize their exploratory movements to serve haptic perception (Drewing, 2014; Ernst & Banks, 2002; Kaim & Drewing, 2011). The performance of the ring finger was lower as compared with index and middle fingers, but higher as compared with the little finger. This nicely parallels its medium frequency of usage (about 30%) found in Experiment 1.

But why do fingers differ in perceptual performance? The number of indentations was held constant in all conditions. However, peak force and finger width differed, yielding potential differences in gathering sensory information. It has been previously suggested that with more deformation of a finger and an object’s surface (e.g., by applying more force), and with a larger contact area, more sensory information on softness can be gathered (Kaim & Drewing, 2011; Nicholson, Maher, & Adams, 1998, Srinivasan & LaMotte, 1995).

Indeed, observations on peak indentation forces parallel the results in JNDs. Higher forces occur more often with lower JNDs: Participants used higher peak force when exploring with the index and middle fingers as compared with the ring and little fingers. During exploration with the little finger the least force was used. Those differences in peak forces are in line with previous measurements (McDermid et al., 2004; Li, Latash, & Zatsiorsky, 1998; Talsania & Kozin, 1998; Quaine, Vigouroux, & Martin, 2003), suggesting that the index and middle fingers are stronger than the other fingers and hence better able to produce high peak force.

Analysis of fingertip width partly paralleled the performance results. Index and middle fingers were wider as compared with the ring finger, and the little finger was the smallest. We used finger width as an indicator for contact area, which is known to be an important factor when exploring compliant objects (Bergmann Tiest, & Kappers, 2009; Srinivasan & LaMotte, 1995). Given the results in Experiment 2, we suggest that differences in perceptual performance are based on at least one of the two factors but cannot clearly distinguish between the influences of these two potential factors. In Experiment 3, we constrained the forces used by each exploring finger to an equal and low level to investigate the sensory sensitivity of the different fingers without the influence of different abilities in force production.

## Experiment 3

In the third experiment we investigated if fingers differ in their sensory sensitivity to softness by systematically comparing the perceptual performance under similar exploration force. In this case only differences in sensory sensitivity influence performance, while differences in finger strength should not influence performance. The results of Experiments 2 and 3 combined therefore allow us to dissociate the influences of force and sensory sensitivity. As in Experiment 2, we used a 2IFC discrimination task to compare softness discrimination using different fingers (index, middle, ring, or little finger). Participants were allowed to indent the surface of an object once per trial. In contrast to Experiment 2, the amount of force used was fixed. Again, as in Experiment 2, we calculated discrimination thresholds (JNDs) for each finger and measured finger tip widths.

### Methods

Sixteen healthy students from the Justus-Liebig-University in Giessen participated in this study (11 females, fivev males, average age 23 years, range: 20–28). All participants reported being right-handed and had normal or corrected-to normal vision. Inclusion conditions were the same as those for *Experiment* 2. Participants were naïve to the purpose of the study, were paid for participating, and gave written informed consent.

Stimuli, setup, procedure, and number and order of trials were almost the same as in Experiment 2. Experiment 3 also included four conditions in which we assessed JNDs for index, middle, ring, and little finger exploration separately. In contrast to Experiment 2, we prescribed constant peak forces during the exploration. Participants were instructed to indent the stimuli with a peak force between 10.0 and 15.0 N; the upper limit was chosen to be close to the average force of the little finger in Experiment 2 (14.8 N) in order to prevent fatigue. In each trial, a mild constant beep sound was presented if the participants applied a force between 10 and 15 N (~900 Hz). In trials where participants did not apply enough force no sound appeared and when participants applied too much force they heard two beep sounds. Participants performed eight test trials in the beginning of the experiment to train precise peak force application within the prescribed range and to get familiar with the setup. Data analyses were similar to those for Experiment 2 with the exception that we only included data from trials with peak indentation forces between 9 and 16 N; a stricter exclusion criterion would have left too few trials for analysis.

### Results

In total 89.3% of the trials were valid and could be used in the analysis (index finger: 88.3%; middle finger: 90.7%; ring finger: 88.0%; little finger: 90.0%). Fingers did not differ in the percentage of valid trials, *F*(3,45) = 0.28, *p* = .840, *η*_*p*_^*2*^ = 0.018. As we would expect, in the ANOVA on averaged PSEs no significant main effect was found, *F*(3,45) = 0.37, *p* = .778, *η*_*p*_^*2*^ = 0.024 (overall average: 0.404 mm/N).

**Fig. 6:**
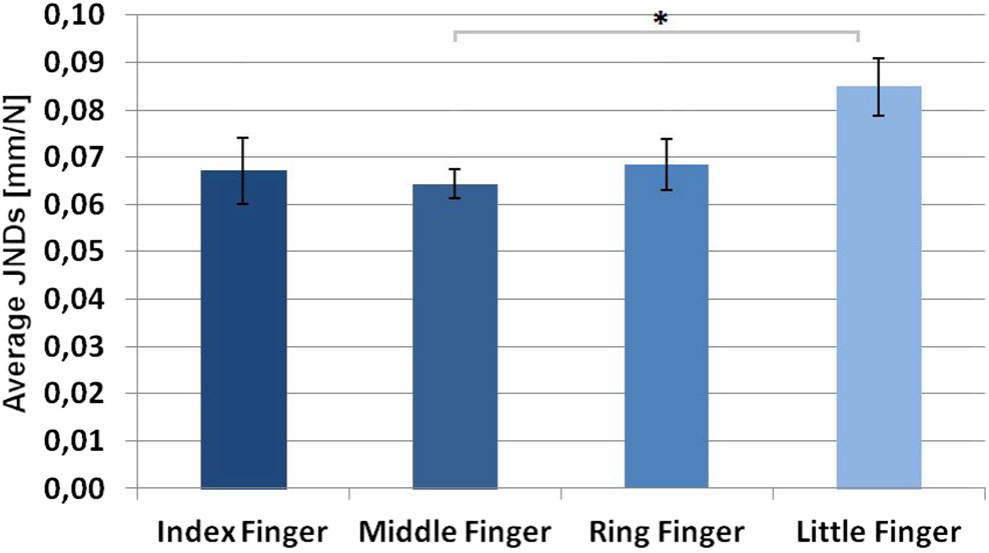
Average just noticeable differences (JNDs; mm/N) and *SEM* for fingers. Significant effects are indicated with an asterisk (* = *p* < .05; ** = *p* < .01)

JNDs significantly differed between fingers (Fig. 6), *F*(3,45) = 4.97, *p* = .005, *η*_*p*_^*2*^ = 0.249. In the Holm-Bonferroni-corrected t-tests we found a significantly higher performance with the middle finger compared with the little finger, *t*(15) = 3.42, *p*_*holm*_ = .023, *d* = 0.85. We found no performance differences between all other finger combinations *–* index and ring finger: *t*(15) = 0.25, *p*_*holm*_ = 1.000, *d* = 0.06, middle and ring finger: *t*(15) = 0.94, *p*_*holm*_ = 1.000, *d* = .23; index and middle finger, *t*(15) = 0.55, *p*_*holm*_ = 1.000, *d* = 0.14; index and little finger, *t*(15) = 2.33, *p*_*holm*_ = .136, *d* = 0.58, ring and little finger *t*(15) = 2.54, *p*_*holm*_ = .113, *d* = 0.64.

**Fig. 7:**
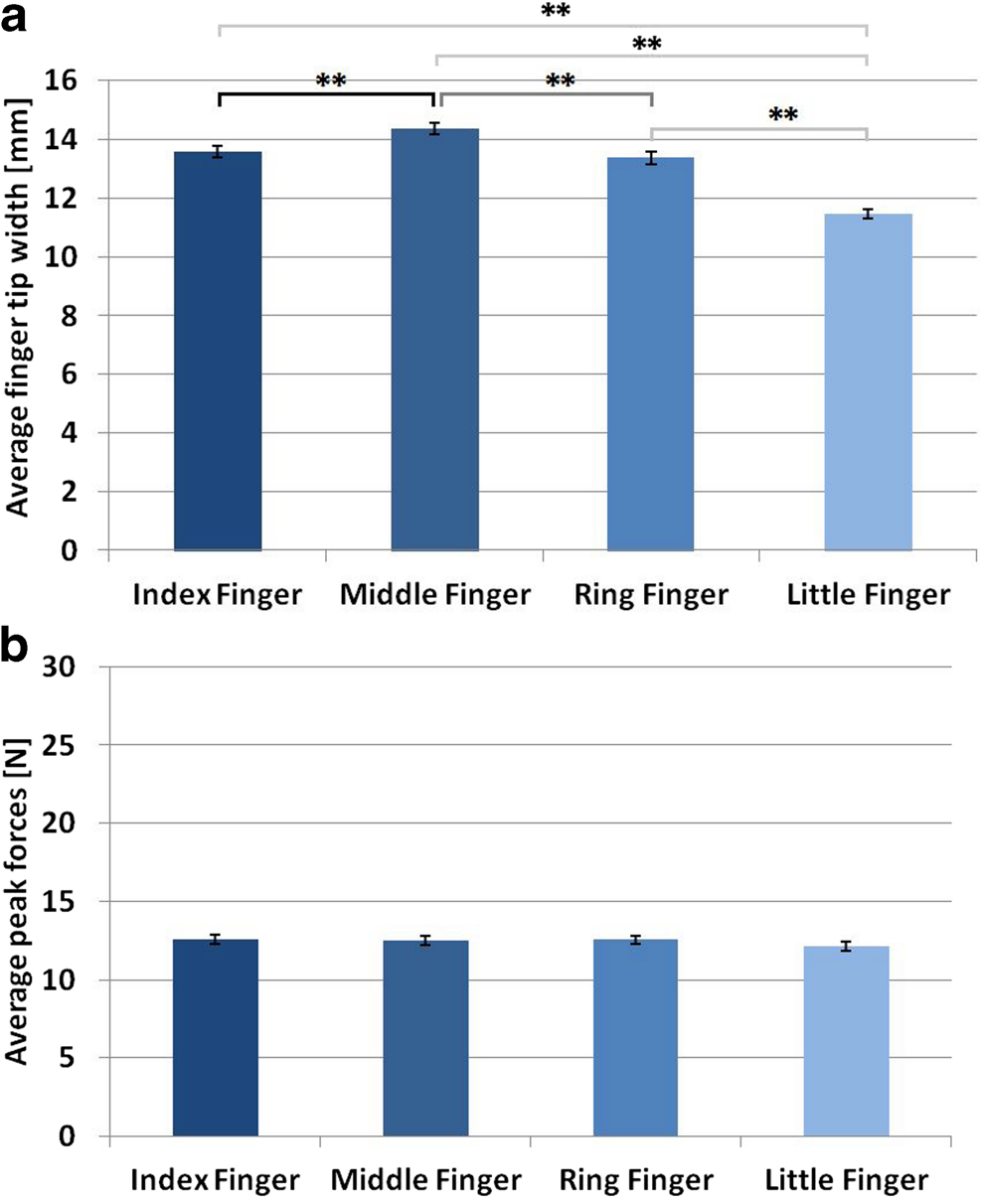
(**A**) Average finger width (mm) and *SEM* for each of the four fingers. (**B**) Average forces (N) and *SEM* for each of the four fingers. Significant effects are indicated by asterisks (* = *p* < .05; ** = *p* < .01)

Fingertips differed significantly in their width, *F*(3,45) = 95.58, *p* < .001, *η*_*p*_^*2*^ = 0.864 (Fig. 7A). Pair-wise *t*-tests with Holm-Bonferroni-corrected p-values showed that the middle finger was on average wider as compared with index, ring, and little fingers, *t*(15) = 5.17, *p*_*holm*_ < .001, *d* = 1.29, *t*(15) = 6.34, *p*_*holm*_ < .001, *d* = 1.59, and *t*(15) = 14.69, *p*_*holm*_ < .001, *d* = 3.67, respectively. The index finger and the ring finger were on average wider as compared with the little finger, *t*(15) = 13.41, *p*_*holm*_ < .001, *d* = 3.35, and, *t*(15) = 9.98, *p*_*holm*_ < .001, *d* = 2.50, respectively. Index and ring fingers did not differ significantly from each other, *t*(15) = 1.04, *p*_*holm*_ = .315, *d* = 0.26.

As expected, we found no significant effect in the ANOVA comparing individually averaged peak forces between different fingers, *F*(3,45) = 1.90, *p* = .144, *η*_*p*_^*2*^ = 0.112. This confirmed that participants used equal forces with each finger (Fig. 7B).

### Discussion

When constant forces were prescribed, participants discriminated softness similarly with the index, middle, and ring fingers, but worse with the little finger. This remaining difference in perceptual performance can be traced back to differences in sensory sensitivity – that is, to lower sensitivity for the little finger *–* given that exploratory movements were highly similar across fingers. Differences in performance of index, middle, and ring fingers in Experiment 2 can now clearly be attributed to differences in force production.

It should be noted that the overall performance of all fingers was worse in Experiment 3 as compared with Experiment 2. Peak forces in Experiment 2 were also higher (and thus perhaps more natural) for each finger as compared with Experiment 3. This finding provides a confirmation of the importance of executed force for softness discrimination performance.

A lower sensitivity for the little finger can explain why participants avoid the use of the little finger in spontaneous exploration (Exp. 1 and Katz, 1925). Additionally, the smaller difference in performance between index, middle, and little fingers in Experiment 3 as compared with Experiment 2 indicates the influence of finger strength on performance differences. Therefore, a more frequent usage of index and middle fingers as compared with the little finger, indicated in Experiment 1, seems to be based on both the ability to produce force and sensory differences. The more frequent usage of index and middle fingers as compared with the ring finger can be explained sufficiently by differences in the ability to produce force, as perceptual performance of the ring finger differed from that of the index and middle fingers in Experiment 2 when spontaneous forces were used, but not when constant forces were used in Experiment 3.

## General discussion

In spontaneous softness discrimination (Exp. 1) participants most frequently used combinations of index, middle, and, to a lesser extent, ring fingers to explore the given stimuli. Alternating between indenting with single fingers and with multiple fingers was a prominent behavior. Preferred fingers were the index finger, then middle finger, then the ring finger. Little finger and thumb were used least frequently. These preferences are paralleled by performance differences (Exp. 2): participants performed better with the index and middle fingers as compared with the ring and little fingers, and better with the ring finger as compared with the little finger. We conclude that finger preferences can well be explained by the aim to select exploratory movements that optimize haptic perception. Comparing the performance with unconstrained finger forces (Exp. 2) to that with prescribed forces (Exp. 3) further showed that performance differences between the fingers are based on both different motor abilities to produce force, mainly linked to using the index and middle fingers, and different sensory sensitivities, mainly linked to avoiding the little finger.

One reason why the fingers differ in sensory sensitivity could be differences in the number of mechanoreceptors in the fingertip contacting the object during exploration (Dillon, Haynes, & Henneberg, 2001; Gellis & Pool, 1977; Martin & Jessel, 1991). According to our results the index and middle fingers are more sensitive to softness than the ring finger, and the little finger is least sensitive. Results from Valbo and Johansson (1978) and Dillon, Haynes, and Henneberg (2001) indicate an equal density of Merkel discs and Meissner´s corpuscles across fingers within one human. We assume that an even spatial density but varying fingertip size *–* and therefore contact area *–* could explain differences in perceptual performance. Here we observed that little fingers are smallest, followed by the ring, index, and finally middle fingers, so this assumption can partly explain observed differences in sensitivity. These assumptions are also in line with Drewing (2014), who showed that using three fingers simultaneously (larger contact area, more receptors) leads to better softness perception as compared with one finger (smaller contact area, fewer receptors). Additionally, sensory sensitivity across fingers correlates with the cortical representation of fingers in S1 and Brodmann 3b and 1. Ducan and Boynton (2007) found correlations between sensitivity threshold and representation size for the thumb, index, middle, ring, and little fingers indicating a larger cortical representation of the larger, more sensitive fingers.

Furthermore, our assumptions are also in line with previous findings in volume perception (Zhang et al., 2018; Zhang et al., 2019), spatial acuity (Ducan & Boynton, 2007; Manser-Smith et al., 2018; Sathian & Zangaladze,^1996^; Schweizer et al., 2000; Vega-Bermudez & Johnson, 2001) and two-point discrimination (Louis et al., 1984). The previous findings in tactile sensitivity are not completely uniform concerning differences between the individual fingers, but the general pattern shows a decreasing sensitivity from the large index and middle fingers to the smallest little finger. The pattern is also paralleled by the general representation of the fingers. The larger, higher performing index and middle fingers are represented more accurately in length than the ring finger. The little finger is maximally underestimated in length (Longo & Haggard, 2010; Longo & Haggard, 2012). However, the relationship between sensory sensitivity and the number of mechanoreceptors involved needs to be further investigated.

A second factor influencing the performance of different fingers in softness exploration is the ability to produce force. In Experiment 2, we found higher indentation forces for the index and middle fingers as compared with the ring and little fingers, accompanied by lower JNDs for the index and middle fingers as compared with the ring and little fingers, and higher forces produced by the ring finger as compared with the little finger accompanied by a lower JND for the ring finger. However, in Experiment 3, where force was constant, performance was worse for all fingers, and finger differences in performance decreased, indicating a substantial influence of peak force on perceptual performance. The peak forces measured for the different fingers are in line with previous measurements (MacDermid et al., 2004; Li, Latash, & Zatsiorsky, 1998; Talsania & Kozin, 1998; Quaine, Vigouroux, & Martin, 2003), suggesting that the index and middle fingers are stronger than the other fingers. The observed increase in perceptual performance for fingers when they indent the surface of an object more strongly fits with previous suggestions that with more deformation of the finger and an object’s surface (e.g., by applying more force) more sensory information on softness can be gathered (Kaim & Drewing, 2011; Nicholson, Maher, & Adams, 1998; Srinivasan & LaMotte, 1995). For the effects of force on perception, again, the number of mechanoreceptors involved may play a role, because with more force and more stimulus deformation the contact area between finger and surface is increased. Another factor to explain these effects could be that the use of more force produces larger sensory differences between differently compliant stimuli.

Overall, we found that both the ability to produce force and sensory sensitivity shape the performance of single fingers in softness perception, and that performance differences can explain the preferences in natural usage of the fingers. In exploration with multiple fingers, integration of sensory information across fingers is possible (DiLuca, 2011). Information from different fingers should be integrated with different weights according to their performance. Our results indicate that if the integration follows an optimization model (Ernst & Banks, 2002; Drewing & Ernst, 2006; Lezkan, Metzger, & Drewing, 2018), we would expect information from the index and middle fingers to have a greater weight as compared with information gathered from the ring and little fingers. We would also expect information gathered with the little finger to have the smallest weight. However, these hypotheses need to be tested in future studies.

Another factor that may also contribute to usage preferences when multiple fingers are involved is enslaving effects: it has been shown that when humans produce force with single or multiple fingers, fingers not intentionally involved also produce some force (Zantsiorsky, Li, & Latash, 2000). A main mechanism for this effect is the interaction of neuronal structures controlling finger flexion. Peripheral tendon connections and multi-digit extrinsic muscles also seem to influence this behavior (Zantsiorsky, Li, & Latash, 2000). The effect seems to be strongest when using the little finger (ring finger also produces force) and the ring finger (middle finger also produces force). Because we find systematic usage (mainly index and middle fingers) and avoidance patterns of specific fingers (mainly avoidance of the little finger), which are different from the enslaving effect during exploration in Experiment 1, we can conclude that finger usage is not mainly driven by this effect. However, we cannot exclude that the usage of multiple fingers might be partly influenced by enslaving effects.

Finally, the observed behavior might also be influenced by the way we presented the stimuli. For example, the size of the explored surface might influence exploration behavior. Observers might use the little finger and thumb more frequently or even use the palm when exploring very large surfaces without movement constraints. This might be tested in a future study. However, we think the chosen stimuli are still representative for many everyday objects and are well comparable to stimuli used in previous softness exploration studies (e.g., Kaim & Drewing, 2011; Srinivasan & LaMotte, 1995; Saig et al., 2012; Zoeller et al., 2019).

Overall, we conclude that in spontaneous exploration observers prefer combinations of index, middle, and partly ring fingers to explore the softness of objects. This behavior seems to be well chosen, as indicated by improved performance with the spontaneously used fingers. Performance differences between the fingers seems to be based on both the different motor abilities to produce force and different sensory sensitivities. The ability to produce more force seems to enhance the usage of the index, middle, and ring fingers, while a lower sensory sensitivity seems to lead to avoidance of the little finger.

## Footnotes

1 Initially the study was planned with two different standard stimuli to counter habituation. However, after repeatedly measuring the stimulus compliance post data collection, we noticed that the two standard stimuli were indeed equally compliant. All analyses were conducted using post data collection compliance values.
